# ITIH4 alleviates OVA-induced asthma by regulating lung-gut microbiota

**DOI:** 10.1186/s10020-025-01270-x

**Published:** 2025-05-23

**Authors:** Yi-Hsuan Liu, Yueh-Lun Lee, Chia-Li Han, Yu-Chun Lo, Zih-An Liao, Yu-Syuan Shih, Yi-Wen Lin, Syue-Wei Peng, Kang-Yun Lee, Shu-Chuan Ho, Sheng-Ming Wu, Cheng-Wei Lin, Kian Fan Chung, Jer-Hwa Chang, Hsiao-Chi Chuang

**Affiliations:** 1 School of Respiratory Therapy, College of Medicine, Taipei Medical University, 250 Wuxing Street, Taipei, 11031 Taiwan; 2 Department of Microbiology and Immunology, School of Medicine, College of Medicine, Taipei Medical University, Taipei, Taiwan; 3 Master Program in Clinical Genomics and Proteomics, College of Pharmacy, Taipei Medical University, Taipei, Taiwan; 4 The Ph.D. Program for Neural Regenerative Medicine, College of Medical Science and Technology, Taipei Medical University, Taipei, Taiwan; 5 Division of Pulmonary Medicine, Department of Internal Medicine, School of Medicine, College of Medicine, Taipei Medical University, Taipei, Taiwan; 6 Division of Pulmonary Medicine, Department of Internal Medicine, Shuang Ho Hospital, Taipei Medical University, New Taipei City, Taiwan; 7 Graduate Institute of Medical Sciences, College of Medicine, Taipei Medical University, Taipei, Taiwan; 8 Department of Biochemistry and Molecular Cell Biology, Taipei Medical University, Taipei, Taiwan; 9 National Heart and Lung Institute, Imperial College London, London, UK; 10 Division of Pulmonary Medicine, Department of Internal Medicine, Wan Fang Hospital, Taipei Medical University, Taipei, Taiwan; 11 Cell Physiology and Molecular Image Research Center, Wan Fang Hospital, Taipei Medical University, Taipei, Taiwan

**Keywords:** Airway hyperresponsiveness, Inflammation, Microbiome, Short-chain fatty acid, Th2

## Abstract

**Background:**

Inter-alpha-trypsin inhibitor heavy chain 4 (ITIH4), a Type 2 acute phase protein, is critical for resolving inflammation and promoting tissue repair. While its role in chronic respiratory diseases is recognized, its effects on asthma remain unclear. This study investigated the effects of ITIH4 on the modulation of lung and gut microbiota, the attenuation of allergic inflammation, and the improvement of respiratory outcomes in an asthma mouse model.

**Methods:**

Six-week-old male Balb/c mice were divided into five groups: control, ITIH4, ovalbumin (OVA), and two OVA + ITIH4 treatment groups at different doses. Lung function and oxygen saturation were measured, and bronchoalveolar lavage fluid (BALF) was analyzed for white blood cell counts and cytokines. Lung and gut microbiota were profiled using 16 S rRNA gene sequencing, and short-chain fatty acids (SCFAs) were measured using gas chromatography-mass spectrometry (GC-MS). Proteomic profiling of intestinal tissues was conducted to identify ITIH4-associated signaling pathways.

**Results:**

ITIH4 administration significantly mitigated OVA-induced asthma symptoms by reducing weight loss, airway resistance, and tissue damping (*p* < 0.05). Histological analysis showed decreased airway wall thickening and lung injury scores (*p* < 0.05). ITIH4 also lowered BALF eosinophils and lymphocytes, IgE, and Th2 cytokines (IL-4, IL-5, and IL-13) (*p* < 0.05). ITIH4 treatment modulated microbiome composition, enriching Gram-positive taxa (*Nocardioidaceae* and *Acholeplasmataceae*) and depleting Gram-negative *Helicobacteraceae* (*p* < 0.05). SCFAs correlated with microbiome alterations, notably reduced 4-methylpentanoic acid levels (*p* < 0.05). Proteomic analysis revealed a dose-dependent activation of granzyme A signaling and suppression of metabolic and solute transport pathways.

**Conclusions:**

ITIH4 ameliorates asthma symptoms by modulating lung and gut microbiota, dampening Th2-driven inflammation, and restoring mucosal immune balance. These findings support ITIH4 as a potential candidate for microbiome-targeted asthma therapy.

## Introduction

Asthma, a prevalent chronic inflammatory respiratory disease (Runnstrom et al. 2022), affects approximately 300 million individuals globally, with an additional 100 million cases projected by 2025 (Maciag and Phipatanakul 2020). It remains a major public health concern, contributing to one in every 250 deaths worldwide (Fergeson et al. 2017). Allergic asthma is the most common phenotype of asthma, which accounts for up to 80% of childhood and over 50% of adult asthma cases (Akar-Ghibril et al. 2020). It is characterized by an exaggerated immune response to typically harmless environmental allergens, leading to airway inflammation, bronchoconstriction, and mucus overproduction (Gohal et al. 2024). In allergic individuals, immature dendritic cells in the lungs capture allergens and develop into antigen-presenting cells (Gaurav and Agrawal 2013). These cells display antigen fragments via the Major Histocompatibility Complex class II (MHC II), stimulating naïve T cells to differentiate into T helper type 2 (Th2) cells, which promote an allergic response (Secrist et al. 1993). In addition to APC-mediated Th2 polarization, other immune mechanisms (including type 2 innate lymphoid cells (ILC2 s), mast cells, basophils, eosinophils, and regulatory T cells (Tregs) play crucial roles in driving and modulating allergic asthma. Cytokines such as IL-4, IL-5, and IL-13 are central to these processes, contributing to IgE production, eosinophilic inflammation, and mucus hypersecretion (Gina Report, 2023; Fahy, 2015). Therefore, allergic asthma is a multifaceted disease involving both innate and adaptive immune responses. Conversely, nonallergic asthma, affecting 10–40% of asthma cases (Baos et al. 2018), is characterized by an acute inflammatory response involving cytokines such as IL-1, IL-6, IL-8, and TNF-α, leading to neutrophil infiltration and airway inflammation (Sze et al. 2020).

Dysbiosis refers to imbalances characterized by altered composition, loss of beneficial microbes, and overgrowth of potentially pathogenic bacteria (DeGruttola et al. 2016). Previous studies have highlighted the role of lung microbiota in asthma, showing that adults with asthma have reduced bacterial diversity and dysbiosis in their respiratory microbiome compared to healthy individuals (Valverde-Molina and García-Marcos 2023; Whiteside et al. 2021). Notably, asthmatic lungs have an increased abundance of *Firmicutes* and *Proteobacteria*, along with a reduced presence of *Actinobacteria* and *Bacteroidota*, leading to airway microbiome dysbiosis (Hufnagl et al. 2020). Animal studies further emphasize the lung microbiota’s role in immune development, as germ-free mice display exaggerated lung inflammation, and neonatal mice exhibit strong allergic reactions that subside as bacterial populations mature (Whiteside et al. 2021). Similarly, the gut microbiota, which harbors an estimated 10¹³−10¹⁴ bacterial colonies, plays a crucial role in health (Lozupone et al. 2012). In healthy individuals, it is dominated by *Firmicutes* and *Bacteroidetes* (Zhao et al. 2023c), but asthma patients often show lower gut microbial diversity, particularly a reduction in *Clostridiales* bacteria (Hua et al. 2016). Gut dysbiosis, characterized by altered microbial composition, has been linked to a higher risk of asthma (Abrahamsson et al. 2014).

The “lung-gut axis” highlights the bidirectional communication between the intestinal flora and lung tissues, playing a crucial role in immune regulation and inflammation (Dang and Marsland 2019). Dysbiosis in the gut microbiota can impair the production of short-chain fatty acids (SCFAs) and other microbial metabolites that are essential for maintaining immune homeostasis and lung health (Trompette et al. 2014). SCFAs, produced by gut bacteria, promote the growth of beneficial microbial populations such as Bacteroidetes and support regulatory T cell function, thereby suppressing excessive inflammation (Dumas et al. 2018; Wang et al. 2025). When dysbiosis occurs, it can lead to increased intestinal permeability and translocation of microbial components (Di Vincenzo et al. 2024). A previous study shows that lipopolysaccharides (LPS)-induced dysbiosis triggered toll-like receptor activation and proinflammatory signaling pathways, ultimately exacerbating inflammation (Zhang et al. 2025; Zhao et al. 2023a). Consequently, therapeutic strategies targeting the gut microbiome, such as dietary changes, probiotics, or other interventions, show potential in alleviating asthma symptoms by restoring balance within the lung-gut axis (Sokolowska et al. 2018).

Inter-alpha-trypsin inhibitor heavy chain 4 (ITIH4), a Type 2 acute phase protein (APP), plays a key role in the body’s response to inflammation (Choi-Miura et al. 2000). APPs are proteins whose plasma concentrations fluctuate in reaction to inflammation, either increasing (positive APPs) or decreasing (negative APPs) (Engler 1995). Unlike positive APPs, such as C-reactive protein (CRP), which are rapidly upregulated within hours of infection or injury and serve as early markers of inflammation, negative APPs like ITIH4 are induced later in the inflammatory process (Piñeiro et al. 1999). ITIH4 is involved in resolving inflammation and facilitating tissue repair (Ebersole and Cappelli 2000). It stabilizes the extracellular matrix by interacting with hyaluronic acid, protecting tissues from damage, and inhibiting proteases to prevent excessive tissue degradation. Primarily expressed in the liver, ITIH4 levels rise in the plasma during inflammation, reflecting its role in the later stages of the acute-phase response (Petrey and de la Motte 2014).

Despite its known anti-inflammatory role, the link between ITIH4 and immune mechanisms relevant to allergic asthma, including those mediated by microbiota and epithelial-immune cell interactions, remains underexplored. ITIH4 has been implicated in restoring epithelial barrier integrity and reducing airway inflammation (Chen et al. 2021b), which are crucial in asthma exacerbation often driven by microbiome-associated dysbiosis. The role of ITIH4 in chronic respiratory disease has been explored in our previous studies (Chen et al. 2021b; Lee et al. 2015); however, its effects on the asthmatic response remain unclear. The objective of this study is to investigate ITIH4’s potential in regulating the composition of lung and gut microbiota, with the aim of inhibiting allergic reactions in the respiratory system and providing a therapeutic benefit for asthma.

## Materials and methods

### Animals

Six-week-old male Balb/c mice were purchased from BioLASCO Taiwan Co., Ltd and housed in the Laboratory Animal Center at Taipei Medical University. The animals were maintained under controlled conditions of 22 ± 2 °C, 55% ± 10% relative humidity, and a 12-hour light/dark cycle. The study protocol was approved by the Institutional Animal Care and Use Committee of Taipei Medical University (IACUC Approval No. LAC-2021-0148).

### Experimental design

**Fig. 1:**
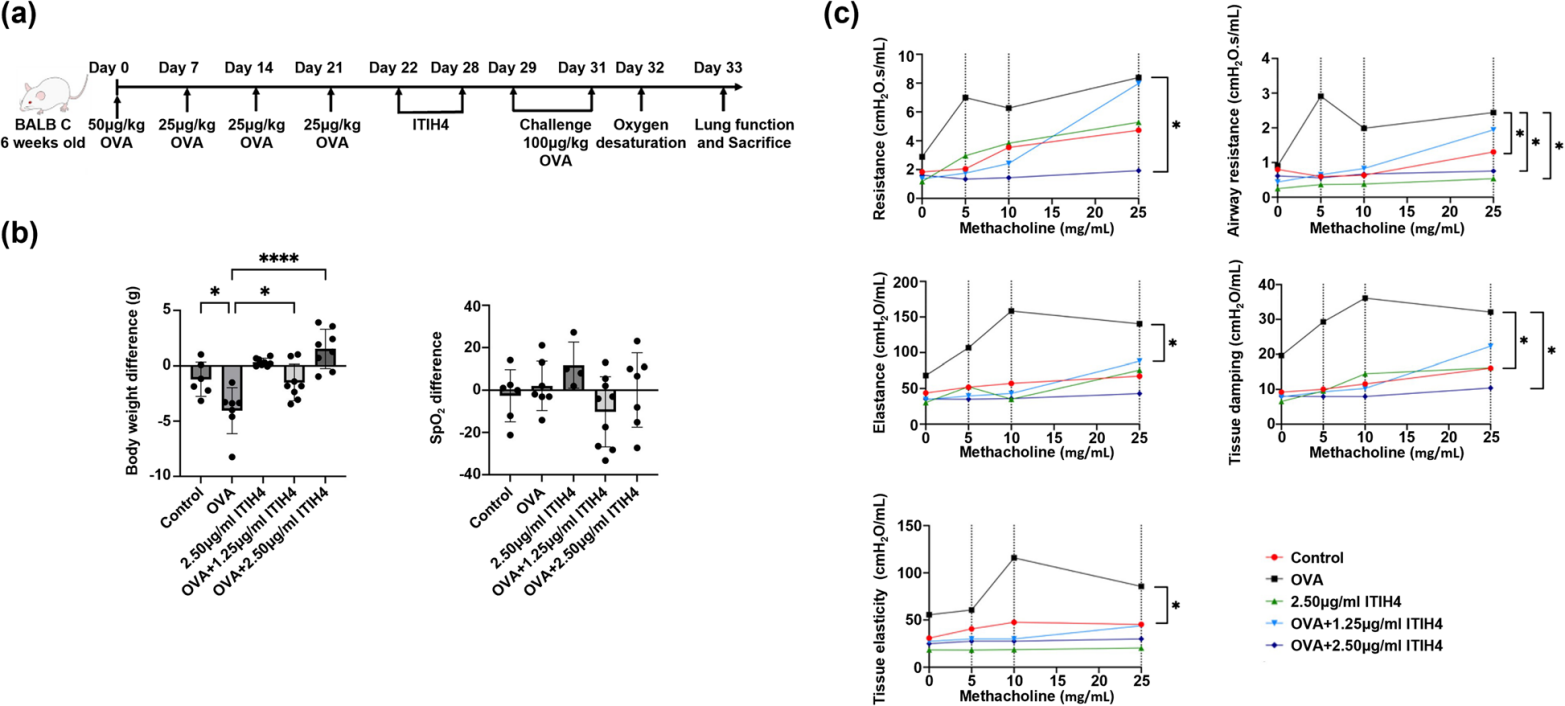
ITIH4 attenuated OVA-induced weight loss and improves lung function in asthmatic mice. **a** Schematic diagram of experiment in which Balb/c mice of 6 weeks old were induced asthma by intraperitoneal administration of OVA followed by intranasal administration of ITIH4. **b** Body weight difference and exercise SpO2 difference in mice. **c** Resistance, airway resistance, elastance, tissue damping, and tissue elastance of mice followed methacholine challenges. *p < 0.05; ****p < 0.0001

Figure 1a illustrates the experimental design. The asthma model induced by ovalbumin (OVA) has been reported previously (Chuang et al. 2013, 2024). Mice were randomly assigned into five groups: control (*n* = 8), ITIH4 (*n* = 8), OVA (*n* = 8), OVA + 1.25 µg/ml ITIH4 (0.203 µg/kg in human; *n* = 8), and OVA + 2.50 µg/ml ITIH4 (0.405 µg/kg in human; *n* = 8). To investigate the therapeutic potential of ITIH4, mice were sensitized with OVA once per week for four weeks to establish an asthma model, followed by ITIH4 treatment. Firstly, mice in the OVA, OVA + 1.25 µg/ml ITIH4, and OVA + 2.50 µg/ml ITIH4 groups received intraperitoneal (*i.p.*) injections of 50 µg/kg OVA (Sigma, St. Louis, MO, USA) on day 0, followed by 25 µg/kg OVA on days 7, 14, and 21. Simultaneously, the control and ITIH4 groups received an equivalent volume of phosphate-buffered saline (PBS) under the same conditions. On day 22, mice in the OVA groups were administered human recommended ITIH4 (CUSABIO, Wuhan, Hubei, China) intranasally (*i.n.*) at concentrations of 0 µg/ml (OVA group), 1.25 µg/ml (OVA + 1.25 µg/ml ITIH4 group), or 2.50 µg/ml (OVA + 2.50 µg/ml ITIH4 group) for continuously 7 days. Mice in the ITIH4 group received only 2.50 µg/ml ITIH4 for 7 days. On day 29, all mice were intranasally administered 100 µg/kg OVA for 3 consecutive days. Intranasal administration was selected to deliver ITIH4 directly to the respiratory tract while minimizing invasive effects, as compared to intratracheal instillation. On day 32, oxygen saturation was measured, followed by lung function tests, and the mice were sacrificed on day 33.

### Oxygen saturation

Oxygen saturation (SpO_2_) was measured using a tissue spectrometer (BIOPAC System, Santa Barbara, USA) with a noninvasive probe attached to the tail of each mouse. Oxyhemoglobin levels were quantified by measuring the absorption of near-infrared light at wavelengths between 800 and 1000 nm, while deoxyhemoglobin concentrations were determined based on the absorption of wavelengths from 600 to 800 nm. Each measurement was conducted over a one-minute period with precise positioning of the probe. Data were analyzed using Biopac Student Lab-BSL software (Upwards Biosystems, Ltd., Taipei, Taiwan).

### Pulmonary function measurement

Pulmonary function was evaluated using the FlexiVent system (SCIREQ, Sterling, VA, USA), which was calibrated according to the manufacturer’s instructions prior to the experiment. A 24-gauge soft catheter was inserted into the trachea to connect the mice to the ventilator in the FlexiVent system. Methacholine at concentrations of 0, 5, 10, and 25 mg/ml was introduced to the lungs via an integrated nebulizer. Respiratory mechanics dose-response curves were generated using both the single-compartment model (single-frequency forced oscillations) and the constant-phase model (multiple-frequency forced oscillations). FlexiWare 8.2.0 software (SCIREQ) was used to apply multiple linear regression analyses to the pressure and volume datasets for each mouse. The following parameters were obtained: total resistance, airway resistance, elastance, tissue damping, and tissue elastance.

### Hematology

Bronchoalveolar lavage fluid (BALF) was collected followed by centrifugation at 1500 rpm for 15 min at 4 °C. The cell pellets were resuspended in PBS, and the total counts of white blood cells, neutrophils, lymphocytes, eosinophils, and monocytes in the BALF were determined using a hematology analyzer (IDEXX Laboratories, Westbrook, Maine, USA).

### Enzyme-linked immunosorbent assay (ELISA)

Enzyme-linked immunosorbent assay (ELISA) was used to quantify various biomarkers in both serum and BALF. Serum IgE levels were measured using ELISA kits from BioLegend (San Diego, USA). In BALF, the levels of interleukin-4 (IL-4), interleukin-5 (IL-5), and tumor necrosis factor-α (TNF-α) were also assessed using BioLegend ELISA kits. Additionally, interleukin-13 (IL-13) levels in BALF were measured using ELISA kits from Thermo Fisher (Waltham, MA, USA). The levels of IL-4, IL-5, and IL-13 were similarly measured in intestinal lysates using the corresponding ELISA kits. All ELISA analyses were performed according to the manufacturer’s instructions.

### Hematoxylin and Eosin (H&E) stains

Lung sections were fixed with 10% buffered formalin via tracheal instillation at a pressure of 25 cmH_2_O for 10 min. After fixation, the lung tissues were embedded in paraffin, sectioned, and stained with hematoxylin and eosin (H&E). Images of the H&E-stained lung sections were captured using the Motic EasyScan Pro system and analyzed with Motic DSAssistant software (Motic, Xiamen, Fujian, China).

### Lung injury assessment

Lung damage was assessed using the K-means clustering algorithm in ImageJ software (National Institute of Health, Bethesda, MD, USA) as described in previous studies (Chen et al. 2021a; Liberti et al. 2021). The K-means clustering algorithm was applied to rank and categorize points on H&E-stained lung slides based on staining intensity and density, corresponding to the severity of the injury (Laiman et al. 2024). Tissue image pixels were grouped into four clusters: background (black), severe damage zones (red), regions of active morphological remodeling as mild damage zones (green), and homeostatic regions as normal zones (blue). To ensure consistency and reduce bias in analyzing heterogeneously injured tissues, each image was compared to a reference image of a single lung lobe and clustered collectively.

### Microbiome DNA extraction and analysis

Microbiome DNA extraction and analysis has been reported previously (Laiman et al. 2024). Bacterial DNA was extracted from lungs and intestines using the QIAamp DNA Stool Mini Kit (QIAGEN, Germany) accordingly to the manufacturer’s instructions. DNA samples with a minimum concentration of 5 ng/µL were stored at −80 °C. The universal 16 S rRNA gene was amplified using V3 (341 F) and V4 (805R) primers, which included Illumina overhang adapters. A finite-cycle PCR amplified the V3-V4 region, and the sequencing libraries were verified with a QSep100 analyzer. Paired-end 300-base reads were generated on an Illumina MiSeq platform. After sequencing, low-quality reads and primers were removed. The DADA2 package (version 1.6) was used for filtering, trimming, and de-noising reads. Chimeras were excluded, and amplicon sequence variants (ASVs) were classified using the SILVA database (version 132) with an 80% confidence threshold. Multiple sequence alignment was done with DECIPHER (version 2.6.0), and a phylogenetic tree was built using RAxML (version 8.2.11). Phyloseq (version 1.22.3) was then used for bacterial community analysis.

### Brown and Brenn gram stain

The tissue sections were deparaffinized in xylene, rehydrated through a graded ethanol series (100%, 95%, 70%, and 50%) for two minutes each, and washed in distilled water. Staining began with Crystal Violet (30 s) followed by rinsing in distilled water. Slides were treated with Gram Iodine Solution (one minute), rinsed, and decolorized in a 1:1 acetone-alcohol solution. They were then counterstained with Basic Fuchsin, rinsed, and immersed in acetone. Staining was enhanced with Picric Acid-Acetone, differentiated in acetone-xylene, cleared in xylene, and mounted. Gram-positive bacteria appeared blue, Gram-negative red, and the background yellow, allowing clear differentiation.

### SCFAs analysis

To extract metabolites from 200 µL of plasma, mix the plasma with 70 µL of water, 50 µL of 20 mM NaOH, and 160 µL of chloroform. Vigorously shake for 1 min, then centrifuge at 1200 g for 15 min. Collect the upper layer, and to 200 µL of this sample, add 70 µL of internal standard (C4-d8), 80 µL of isobutanol, and 100 µL of pyridine. Shake thoroughly, then add 50 µL of isobutyl chloroformate, shaking for an additional 2 min, followed by 5 min of sonication for esterification. Afterward, add 100 µL of hexane, shake, and centrifuge at 1200 g for 5 min. Collect the upper layer for analysis. SCFA analysis was performed using a Bruker GC-MS system (Bruker 436GC coupled with Bruker EVOQ Mass Spectrometer) controlled by Bruker MSWS 8.2 software. Separations were carried out on a VF-5 ms column (30 m × 0.25 mm, 0.5 μm) with helium as the carrier gas at a flow rate of 1.0 mL/min. The oven temperature was held at 40 °C for 5 min, then increased to 310 °C at 10 °C/min. Injection volumes for samples and standards were 2.0 µL with a split ratio of 50:1. The inlet, transfer line, and ion source temperatures were 260 °C, 280 °C, and 250 °C, respectively, with electron energy at 70 eV. Total SCFA concentration was calculated as the sum of formic acid, acetic acid, propionic acid, butyric acid, isobutyric acid, pentanoic acid, 3-methylbutanoic acid, 2-methylbutanoic acid, 3-methylpentanoic acid, and 4-methylpentanoic acid.

### Proteomic analysis

The intestine tissue lysate from individual mouse was subjected to hemoglobin removal by using Hemoglobind (Biotech Support Group LLC, NJ, USA). The depleted lysates from the same group were pooled and then performed tryptic digestion using S-Trap protocol (Protifi, NY, USA) (Zougman et al. 2014). The resulting peptides from control, OVA + 1.25 µg/ml ITIH4, OVA + 2.5 µg/ml ITIH4, and OVA groups were labeled with tandem mass tag (TMT) 126, TMT 128, TMT 129, TMT 131, respectively. The TMT-labeled peptides were combined for peptide fractionation using Pierce™ high pH reversed-phase peptide fractionation kit (ThermoFisher Scientific) following manufacturer’s instruction. Eight fractions were collected for subsequent liquid chromatography tandem mass spectrometry analysis as described in our previous study (Chang et al. 2022). Proteome identification was obtained by searching the raw data against the SwissProt mouse protein database (vers. 2024.06, 17,212 sequences) using MaxQuant (vers. 2.1.4.0). A false discovery rate of 1% was applied to peptide-spectral-match and protein levels to filter confident peptide and protein identifications. Proteome quantification was achieved by estimating the intensity of unique peptides followed with normalization by the total protein abundance. Proteins with 1.3-fold changes in abundance (log2 ratio of > 0.38 or < −0.38) were considered differentially expressed proteins. The pathway analysis was generated through the use of QIAGEN IPA (QIAGEN Inc., https://digitalinsights.qiagen.com/IPA).

### Statistical analysis

Data are presented as mean ± standard deviation (SD). Analysis of variance (ANOVA) with Tukey’s post-hoc test was performed for multiple-group comparisons. Simple linear regression was applied to assess lung function tests. Alpha diversity metrics were calculated using the estimated richness function in the phyloseq package, and beta diversity was evaluated through unweighted UniFrac principal coordinate analysis (PCoA). Pearson’s correlation was used to examine the relationships between (1) lung and intestinal microbiota, (2) lung microbiota and short-chain fatty acids, and (3) fecal microbiota and short-chain fatty acids. All data analyses were performed using GraphPad (version 10, San Diego, CA, USA), with statistical significance set at *p* < 0.05.

## Results

### ITIH4 mitigated OVA-induced weight loss and improves pulmonary mechanics

Administration of ITIH4 effectively alleviated the weight loss typically induced by OVA sensitization, with treated mice maintaining significantly better body weight profiles compared to the OVA-only group (*p* < 0.05; Fig. 1b). Improvements in lung function were also observed, as ITIH4 significantly reduced the elevated airway resistance and tissue damping seen in OVA-induced asthma mice (*p* < 0.05; Fig. 1c). These findings indicate a protective effect of ITIH4 on pulmonary mechanics. However, no notable differences in SpO₂ were observed among the groups, suggesting that the improvements were localized to mechanical function rather than gas exchange.

### ITIH4 reduced airway wall thickening, lung injury, and inflammatory cell infiltration

**Fig. 2:**
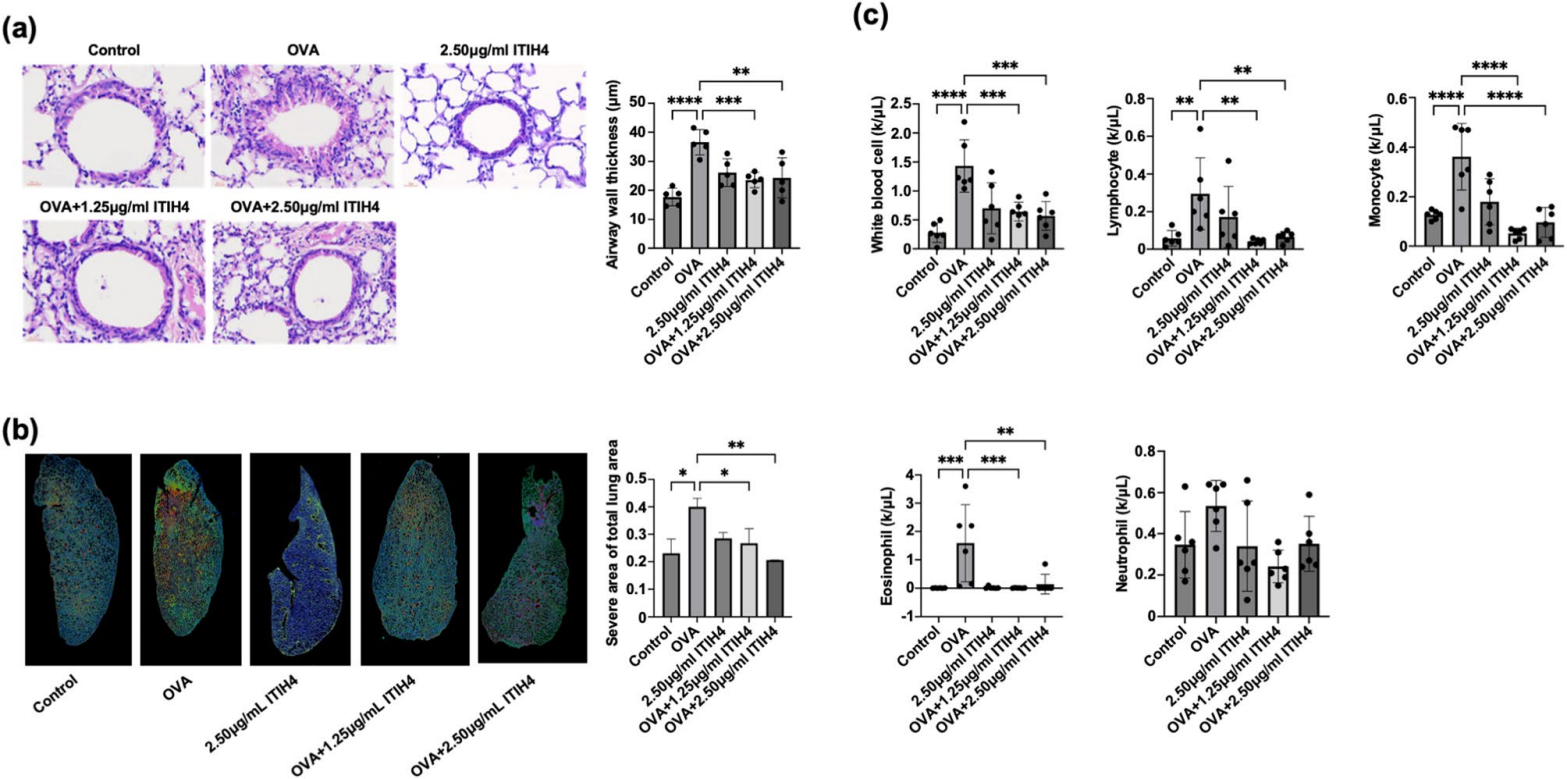
ITIH4 reduced airway wall thickening, lung injury, and inflammatory cell infiltration. **a** Airway wall thickness of OVA-induced asthma mice with ITIH4 administration. **b** Lung damage severity assessment by the k-means clustering algorithm. The image depicts regions (blue) that were classified as normal zones, active morphological remodeling (green) areas as mild damage zones, and places of maximum damage (red) defined as severe damage zones. **c** Total number of BALF white blood cell, lymphocyte, monocyte, eosinophil and neutrophil of OVA-induced asthma mice with ITIH4 administration. * *p* < 0.05; ** *p* < 0.01; *** *p* < 0.001; **** *p* < 0.0001

Histological examination demonstrated that ITIH4 treatment significantly reduced airway wall thickening and the severity of lung tissue damage in OVA-induced asthma mice (*p* < 0.05; Fig. 2a and b). This histopathological improvement was accompanied by a marked decrease in BALF cellular infiltration, including total white blood cells, lymphocytes, monocytes, and eosinophils (*p* < 0.05; Fig. 2c). Notably, mice treated with the higher dose of ITIH4 did not exhibit significant histological differences from control animals, supporting the safety and tolerability of high-dose ITIH4.

### ITIH4 suppressed IgE and Th2 cytokines in lung and intestine

**Fig. 3:**
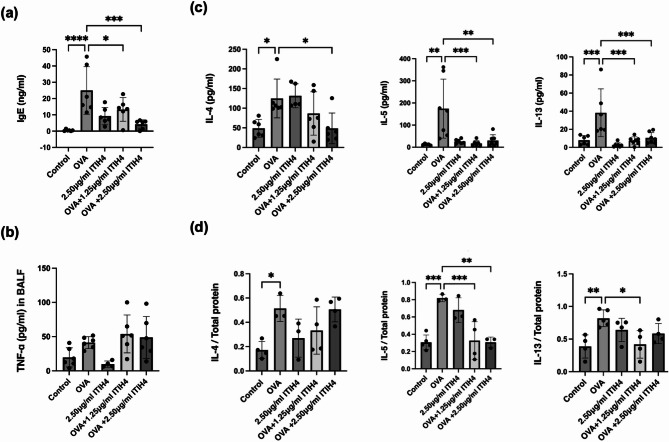
ITIH4 suppressed IgE and Th2 cytokines in serum, BALF, and intestinal tissues. **a** Serum IgE, **b** BALF TNF-α, **c** BALF IL-4, IL-5 and IL-13, and **d** intestine IL-4, IL-5 and IL-13 in OVA-induced asthma mice with ITIH4 administration. * *p* < 0.05; ** *p* < 0.01; *** *p* < 0.001; **** *p* < 0.0001

Systemic allergic sensitization, as reflected by elevated total IgE levels, was significantly diminished in ITIH4-treated mice (*p* < 0.05; Fig. 3a). Although TNF-α levels remained unchanged (Fig. 3b), Th2 cytokines associated with allergic asthma (i.e. IL-4, IL-5, and IL-13) were substantially reduced in BALF following ITIH4 administration (*p* < 0.05; Fig. 3c). Furthermore, ITIH4 also suppressed IL-5 and IL-13 levels in the intestinal tissues of OVA-induced asthma mice (*p* < 0.05; Fig. 3d), suggesting a systemic immunomodulatory effect that extends beyond the lungs.

### ITIH4 modulated lung microbiota composition and redistributes gram-positive/negative bacteria

**Fig. 4:**
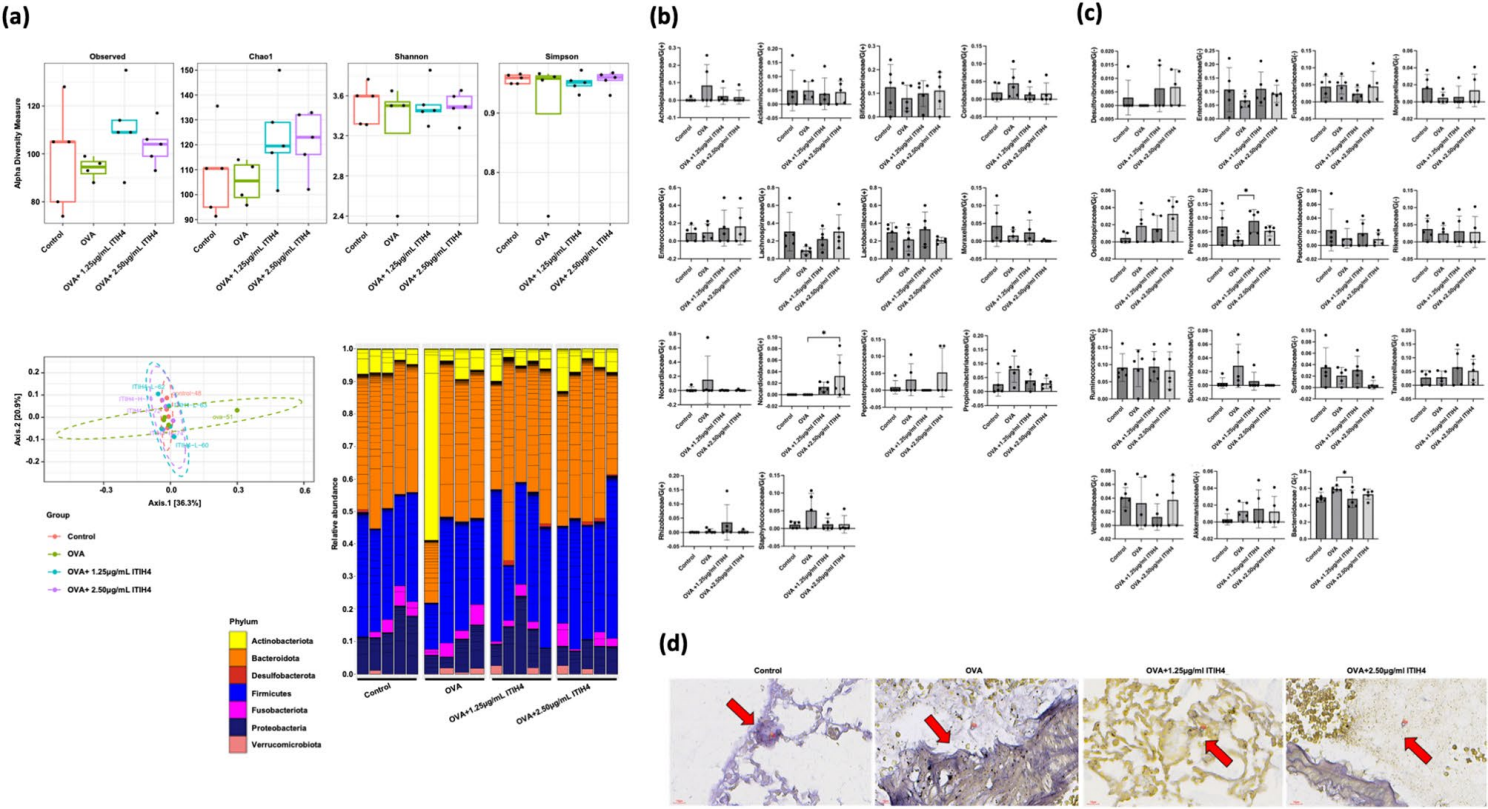
ITIH4 modulated lung microbiota composition and redistributes gram-positive/negative bacteria. **a** Alpha diversity analysis including Observed, Chao1, Shannon, and Simpson indexes. Beta diversity analysis of lung microbiome. **b** Gram-positive bacterial at the phylum level. **c** Gram-negative bacterial at the phylum level. **d** Brown-Brenn tissue gram stain (magnification 80X). The arrows showed Gram-positive bacteria surrounding alveolar regions in controls and OVA groups, and Gram-negative bacteria clustering in alveolar zones of ITIH4-treated lungs. * *p* < 0.05

Microbiome analysis of lung tissues showed that while alpha diversity remained stable, principal coordinate analysis (PCoA) revealed distinct beta diversity clustering between OVA and ITIH4-treated groups, indicating shifts in microbial composition (Fig. 4a). The dominant phyla remained *Bacteroidota* and *Firmicutes*, but ITIH4 treatment led to notable taxonomic changes. Gram-positive *Nocardioidaceae* significantly increased in the high-dose ITIH4 group (*p* < 0.05; Fig. 4b), while Gram-negative *Prevotellaceae* increased in the low-dose group (*p* < 0.05; Fig. 4c). Spatial localization patterns from histological staining further highlighted these changes, with Gram-positive bacteria distributed around alveolar regions in controls and OVA mice, and Gram-negative bacteria predominating in alveolar zones of ITIH4-treated lungs (Fig. 4d).

### ITIH4 reshaped intestinal microbiota and reduces gram-negative bacterial dominance

**Fig. 5:**
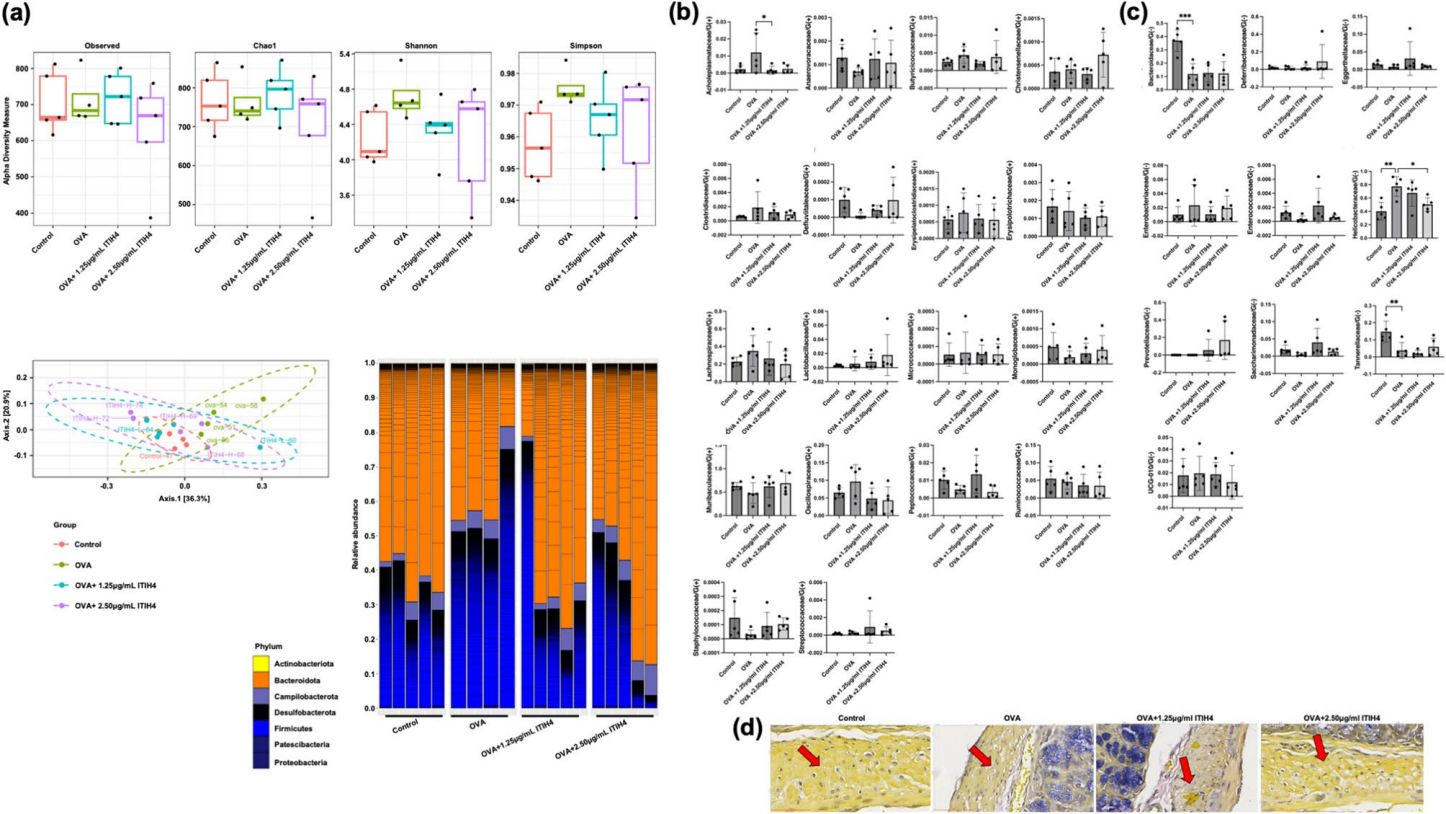
ITIH4 reshaped intestinal microbiota and reduces gram-negative bacterial abundance. **a** Alpha diversity analysis including Observed, Chao1, Shannon, and Simpson indexes. Beta diversity analysis of lung microbiome. **b** Gram-positive bacterial at the phylum level. **c** Gram-negative bacterial at the phylum level. **d** Brown-Brenn tissue gram stain (magnification 80X). The arrows showed Gram-negative bacteria were visualized predominantly along the intestinal mucosa in the ITIH4 groups, indicating compartmental redistribution possibly linked to inflammation control. * *p* < 0.05; ** *p* < 0.01; *** *p* < 0.001

Intestinal microbiota profiling revealed that, although alpha diversity was unaffected, ITIH4 treatment significantly altered beta diversity (*p* < 0.05; Fig. 5a). These changes were characterized by an increase in Gram-positive *Acholeplasmataceae* and a concurrent reduction in Gram-negative *Helicobacteraceae*, the latter of which was elevated in OVA-treated animals (*p* < 0.05; Fig. 5b and c). Microscopy confirmed compartmental shifts, with Gram-negative bacteria localizing predominantly along the mucosal surfaces in ITIH4-treated intestines, potentially reflecting an anti-inflammatory redistribution pattern (Fig. 5c, arrows). 

### ITIH4 modulated SCFA profiles and their correlations with microbial taxa

**Fig. 6:**
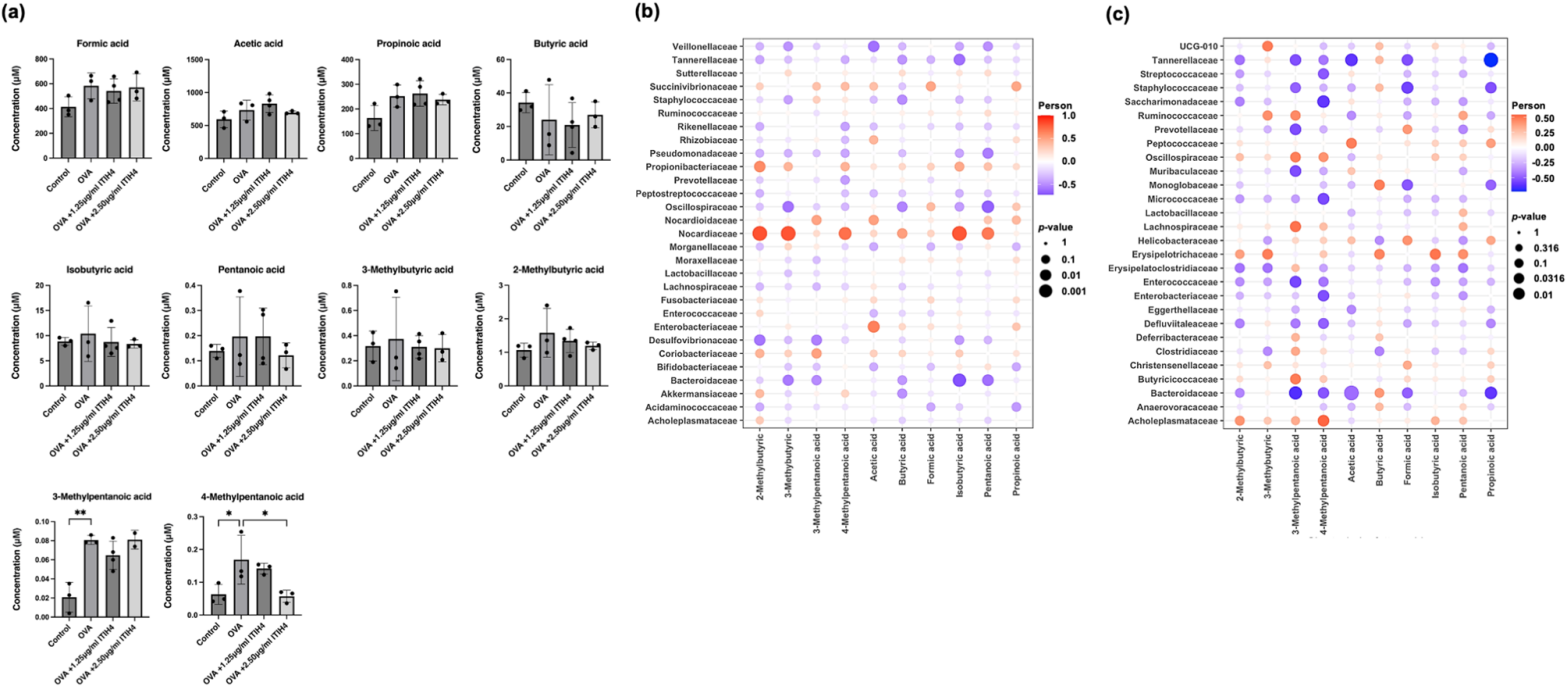
ITIH4 modulated SCFA levels and correlates with microbiome profiles in lung and gut. **a** Serum SCFAs of OVA-induced asthma mice with ITIH4 administration. **b** Correlation of SCFAs with lung microbiome at the family level. **c** Correlation of SCFAs with intestine microbiome at the family level. * *p* < 0.05; ** *p* < 0.01

SCFA analysis demonstrated that ITIH4 significantly reduced the elevated levels of 4-methylpentanoic acid found in OVA-treated mice (*p* < 0.05; Fig. 6a). Correlation matrix analysis revealed that *Nocardioidaceae* positively associated with beneficial SCFAs such as 2-methylbutanoic acid, isobutyric acid, and pentanoic acid (*p* < 0.05; Fig. 6b), while *Bacteroidaceae* showed inverse associations with these metabolites. Similarly, in the intestine, Gram-negative taxa such as *Tannerellaceae* and *Bacteroidaceae* negatively correlated with SCFAs including acetic, formic, and propionic acids (*p* < 0.05; Fig. 6c), indicating SCFA-linked microbial reprogramming driven by ITIH4. 

### ITIH4 induced dose-dependent proteomic reprogramming in intestinal tissues

**Fig. 7:**
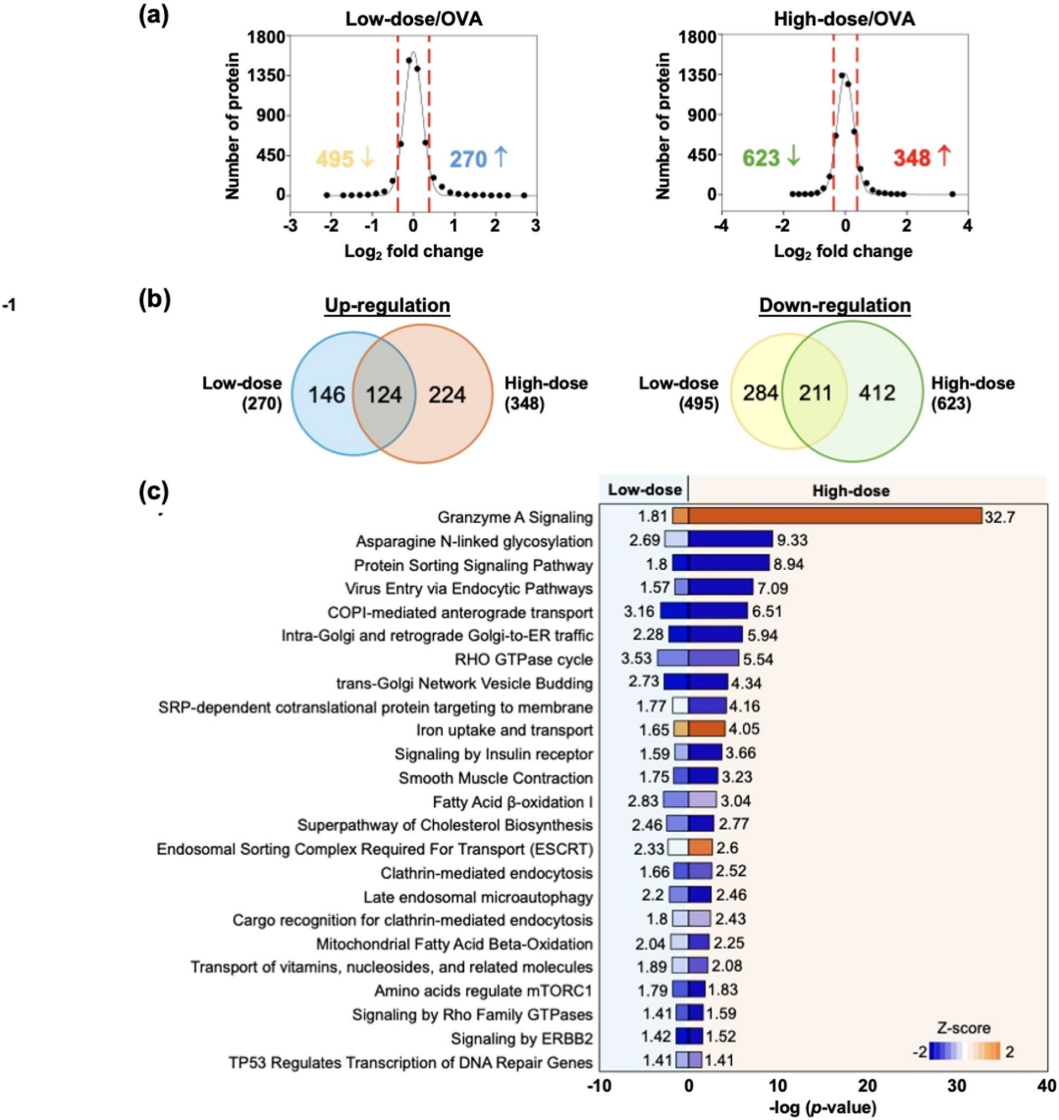
ITIH4 induced dose-dependent proteomic alterations in intestinal tissues. **a** The protein ratio distributions of low-dose and high-dose ITIH4 treatments in compared with untreated control. The red dash lines indicate the threshold of up- or down-regulation. **b** Overlapping of up- and down-regulated proteins in the high-dose and low-dose ITIH4 groups. **c** The enriched pathways with dose-dependent activation or suppression. Pathway name in blue is related to metabolism of proteins, in green for small molecule metabolism, and in purple for molecular transportations. Pathway with z-score > 0 indicates activation while z-score < 0 indicates suppression

Proteomic analysis of intestinal tissues identified 4,724 proteins, with 971 and 765 differentially expressed proteins in the low- and high-dose ITIH4 groups, respectively (Fig. 7a). The high-dose group exhibited a stronger suppressive effect on OVA-induced proteomic disturbances. Approximately 24% and 23% of proteins were commonly regulated between the two doses (Fig. 7b), indicating core conserved responses. Functional pathway enrichment highlighted the activation of granzyme A signaling, iron transport, and endosomal trafficking, along with suppression of key metabolic pathways, including asparagine N-linked glycosylation, RHO GTPase cycling, insulin receptor signaling, and various protein and small molecule transport mechanisms (Fig. 7c). These findings reveal multifaceted molecular regulation by ITIH4, underscoring its systemic therapeutic potential. 

## Discussion

The significance of this study lies in the observation that ITIH4 alleviated OVA-induced airway inflammation and asthma symptoms. The novelty lies in ITIH4’s ability to modulate microbiome dysbiosis, particularly by reducing *Helicobacteraceae*, potentially improving disease outcomes. SCFAs may mediate lung–gut microbiome interactions in asthma. Furthermore, ITIH4 regulated gut immune response, metabolic regulation, and protein processing in OVA-induced asthma models. These findings suggest that ITIH4 may serve as a therapeutic agent by modulating immune responses and microbiome balance.

Our results demonstrated that ITIH4 administration significantly reduced airway wall thickening, lung injury, airway resistance, and elastance, indicating its role in reducing airway remodeling and hyperresponsiveness, major drivers of asthma pathology. ITIH4 also exhibited immunomodulatory effects by reducing white blood cell infiltration, particularly eosinophils, in the BALF, which are central to allergic asthma by driving airway inflammation and hyperreactivity (Holgate 2012). The reduction in eosinophils and Th2 cytokines, such as IL-4, IL-5, and IL-13, supports ITIH4’s role in suppressing the Th2-driven allergic response. These findings suggest that ITIH4 may help reduce the severity of allergic airway inflammation. As a negative APP, ITIH4 protects against proteases that drive tissue damage and inflammation, potentially limiting airway damage and remodeling in asthma progression (Ma et al. 2021). Abnormal levels of ITIH4 have also been observed in chronic obstructive pulmonary disease (COPD) and sepsis, correlating with disease severity (Lee et al. 2015; Zhao et al. 2023b). However, the levels of circulating endogenous ITIH4 were not assessed in this study. Future investigations should explore whether ITIH4 may function not only as a therapeutic agent but also as a potential biomarker for asthma diagnosis or disease monitoring. Together, ITIH4’s protease-inhibiting and immunomodulatory effects may help reduce asthma severity.

Next, we identified that asthmatic mice showed dominant *Bacteroidota* and *Firmicutes*, aligning with previous studies on bronchoalveolar and nasal lavage fluid (Zheng et al. 2021). A prior study suggested that alterations in *Bacteroidetes* and *Firmicutes* might be associated with the development of asthma following air pollution exposure (Valverde-Molina and García-Marcos 2023). Notably, PCoA using weighted UniFrac distance revealed that OVA significantly altered the bacterial distribution away from the control group, whereas ITIH4 administration shifted the microbiota back toward the control group in both the lung and intestine. The results indicate that ITIH4 may mitigate microbiome dysbiosis caused by OVA-induced asthma.

We further identified specific Gram-negative and Gram-positive bacteria affected by ITIH4 administration in OVA-induced asthma in both the lungs and intestines. In the lungs, ITIH4 modulated *Nocardioidaceae* (Gram-positive) and *Prevotellaceae* (Gram-negative), while in the intestine, it affected Gram-positive *Acholeplasmataceae* and notably reduced the abundance of Gram-negative *Helicobacteraceae*. Previous studies have shown that an increased presence of Gram-negative bacteria in sputum was linked to asthma exacerbations in children (Kim et al. 2021) and adults (Ahmed et al. 2020). In our study, *Helicobacteraceae* was elevated in OVA-induced asthma mice, but ITIH4 administration restored it to normal levels. *Helicobacter pylori*, a member of *Helicobacteraceae*, is associated with gastritis, ulcers, and gastric cancer (Kusters et al. 2006). Recent studies also suggest a potential link between *Helicobacter pylori* infection and an increased risk of adult-onset asthma due to its effects on immune responses and inflammation (Wang et al. 2017). However, other reports suggest that Helicobacter pylori may have protective effects against allergic diseases (Liu et al. 2024). In adult asthmatics, the respiratory microbiome often shows reduced diversity and increased bacterial abundance (Valverde-Molina and García-Marcos 2023), suggesting that the presence or absence of *Helicobacteraceae* may play a role in asthma stability. ITIH4’s ability to restore *Helicobacteraceae* levels suggests it may help mitigate intestinal dysbiosis in asthma.

The *Helicobacteraceae* family, including *Helicobacter pylori*, has been associated with disruptions in gut microbial composition, which can affect the production of beneficial metabolites such as SCFAs (Fakharian et al. 2022; Frost et al. 2019). *Helicobacteraceae* primarily utilize nitrogen and carbon sources, relying on amino acids for energy instead of sugars (Kusters et al. 2006). *Helicobacter pylori*, in particular, can metabolize amino acids like leucine and valine, which lead to branched-chain fatty acid (BCFA) production (Zhang et al. 2017). In this study, we observed that BCFAs, including 3-methylpentanoic acid and 4-methylpentanoic acid, were elevated in OVA-induced asthma mice, but ITIH4 treatment at a concentration of 2.50 µg/ml reduced the levels of 4-methylpentanoic acid. SCFAs are key modulators of inflammation and immune function (Ney et al. 2023), which may explain the reduction in OVA-induced IgE and Th2 cytokines following ITIH4 administration, especially at 2.50 µg/ml. 4-methylpentanoic acid, a BCFA derived from branched-chain amino acids such as isoleucine and leucine, is produced by specific protein-fermenting gut bacteria, including members of the Clostridia class and other anaerobic Firmicutes (Reifenberg and Zimmer 2024). These bacteria specialize in proteolytic fermentation, releasing BCFAs as by-products of amino acid metabolism, thereby contributing to gut microbial diversity and host metabolic regulation (Bourdeau-Julien et al. 2023). In our study, BCFAs correlated with *Nocardioidaceae* and *Bacteroidaceae*. In addition, non-BCFAs such as pentanoic acid, acetic acid, formic acid, and propionic acid were correlated with *Nocardioidaceae*, *Bacteroidaceae*, and *Tannerellaceae*. The link between *Helicobacteraceae* and 4-methylpentanoic acid remains poorly understood. While *Helicobacter pylori* may not directly ferment amino acids to produce BCFAs, its presence could modulate the gut microenvironment, impacting amino acid availability and metabolic cross-feeding with other microbes, thereby indirectly influencing BCFA production. Importantly, ITIH4 may influence BCFA metabolism by reshaping gut microbial composition toward less proteolytic, Gram-negative-dominated communities and enhancing the growth of Gram-positive taxa associated with favorable SCFA profiles. However, more evidence is required to examine the BCFA metabolism by ITIH4 in the future.

We identified the key biological processes influenced by ITIH4 in the OVA-induced asthma mice, by activating granzyme A signaling, iron uptake and transport, and the ESCRT. The findings from the intestinal protein expression and pathways of OVA-induced asthma mice may have direct relevance to SCFAs, which regulate gut homeostasis, immunity, and metabolism. SCFAs play a role in immune modulation, affecting T-cell differentiation and inflammation, processes linked to granzyme A signaling and Th2-driven asthma pathology. Granzyme A signaling is critical for immune cytotoxicity (Cigalotto and Martinvalet 2024). A previous reports showed that increased expression of granzyme A in airway was associated with fatal asthma (Annoni et al. 2015). The observed decrease in lymphocyte counts in BALF of ITIH4-treated OVA-induced asthma mouse may partially reflect reduced infiltration or activation of cytotoxic immune cells, particularly CD8⁺ T cells and Natural Killer (NK) cells, which are major sources of Granzyme A at sites of inflammation. This aligns with the observed downregulation of granzyme A signaling, suggesting that ITIH4 may attenuate cytotoxic immune responses in allergic asthma. Also, our results indicate that ITIH4 administration significantly reduced the elevated levels of 4-methylpentanoic acid induced by OVA, suggesting a potential modulatory effect of ITIH4 on SCFA metabolism. SCFAs, influence iron absorption, which aligns with the observed activation of iron uptake pathways in ITIH4-treated mice. It is noticed that the pathophysiologic events producing asthma, including inflammation, increases in Th2 cells, and muscle contraction, are correlated with iron availability (Ghio 2016). ITIH4 activated ESCRT pathways involved in protein trafficking (Verma and Marchese 2015), suggests that ITIH4 may facilitate cellular transport mechanisms that are critical for maintaining intestinal epithelial function.

Conversely, several pathways were suppressed in a dose-dependent manner, including asparagine N-linked glycosylation, RHO GTPase cycle, insulin receptor signaling, and various metabolic processes. Suppressed N-linked glycosylation may disrupt protein folding (Pinho et al. 2023), which could impact cellular communication and immune responses. SCFAs contribute to gut epithelial integrity and intracellular transport, potentially linking them to ITIH4’s effects on ESCRT and RHO GTPase signaling. RHO GTPase signaling, essential for cytoskeleton dynamics and cell motility (Raftopoulou and Hall 2004), was also downregulated, possibly affecting intestinal epithelial dynamics. The observed suppression of insulin receptor signaling may also reflect an SCFA-mediated metabolic shift, as SCFAs are known to regulate glucose metabolism and insulin sensitivity via GPCR signaling. Reduced insulin signaling may reflect shifts in glucose and energy metabolism (Lennicke and Cochemé 2021). These findings suggest a complex interaction between ITIH4, SCFA metabolism, and microbiota composition, influencing both intestinal and immune regulation. Further research is required to clarify these interactions and their potential physiological implications in asthma.

## Conclusions

This study demonstrates that ITIH4 mitigates OVA-induced asthma symptoms by reducing airway inflammation, improving lung function, and modulating immune pathways. In addition, ITIH4 alters the composition of both lung and intestinal microbiota and regulates SCFA metabolism, suggesting its involvement in gut–lung axis interactions and systemic immune modulation. These findings highlight the potential of nasal delivery of ITIH4 as a therapeutic strategy for asthma, particularly in phenotypes associated with microbiome dysbiosis and epithelial barrier dysfunction. The anti-inflammatory and epithelial-protective properties observed in this work support the translational potential of ITIH4 as a novel biologic for asthma therapy.

## Data Availability

The datasets used and/or analyzed during the current study are available from the corresponding author on reasonable request.
