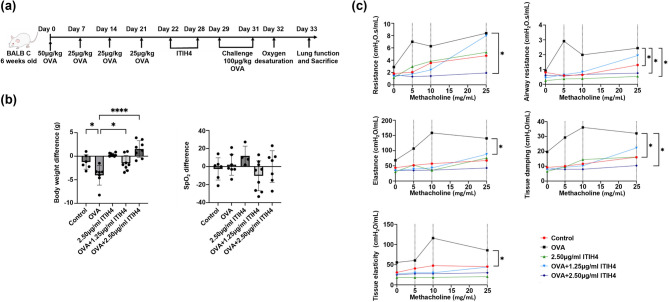# Correction: ITIH4 alleviates OVA-induced asthma by regulating lung-gut microbiota

**DOI:** 10.1186/s10020-025-01293-4

**Published:** 2025-06-25

**Authors:** Yi-Hsuan Liu, Yueh-Lun Lee, Chia-Li Han, Yu-Chun Lo, Zih-An Liao, Yu-Syuan Shih, Yi-Wen Lin, Syue-Wei Peng, Kang-Yun Lee, Shu-Chuan Ho, Sheng-Ming Wu, Cheng-Wei Lin, Kian Fan Chung, Jer-Hwa Chang, Hsiao-Chi Chuang

**Affiliations:** 1 School of Respiratory Therapy, College of Medicine, Taipei Medical University, 250 Wuxing Street, Taipei, 11031 Taiwan; 2 Department of Microbiology and Immunology, School of Medicine, College of Medicine, Taipei Medical University, Taipei, Taiwan; 3 Clinical Genomics and Proteomics, College of Pharmacy, Taipei Medical University, Taipei, Taiwan; 4 Program for Neural Regenerative Medicine, College of Medical Science and Technology, Taipei Medical University, Taipei, Taiwan; 5 Division of Pulmonary Medicine, Department of Internal Medicine, School of Medicine, College of Medicine, Taipei Medical University, Taipei, Taiwan; 6 Division of Pulmonary Medicine, Department of Internal Medicine, Shuang Ho Hospital, Taipei Medical University, New Taipei City, Taiwan; 7 Graduate Institute of Medical Sciences, College of Medicine, Taipei Medical University, Taipei, Taiwan; 8 Department of Biochemistry and Molecular Cell Biology, Taipei Medical University, Taipei, Taiwan; 9 National Heart and Lung Institute, Imperial College London, London, UK; 10 Division of Pulmonary Medicine, Department of Internal Medicine, Wan Fang Hospital, Taipei Medical University, Taipei, Taiwan; 11 Cell Physiology and Molecular Image Research Center, Wan Fang Hospital, Taipei Medical University, Taipei, Taiwan

**Correction: Molecular Medicine 31**,** 204 (2025)**

**https://doi.org/10.1186/s10020-025-01270-x**

In this article (Liu et al. 2025), Fig. 1 appeared incorrectly and have now been corrected in the original publication. For completeness and transparency, the old incorrect versions are displayed below.

Incorrect Fig. 1.


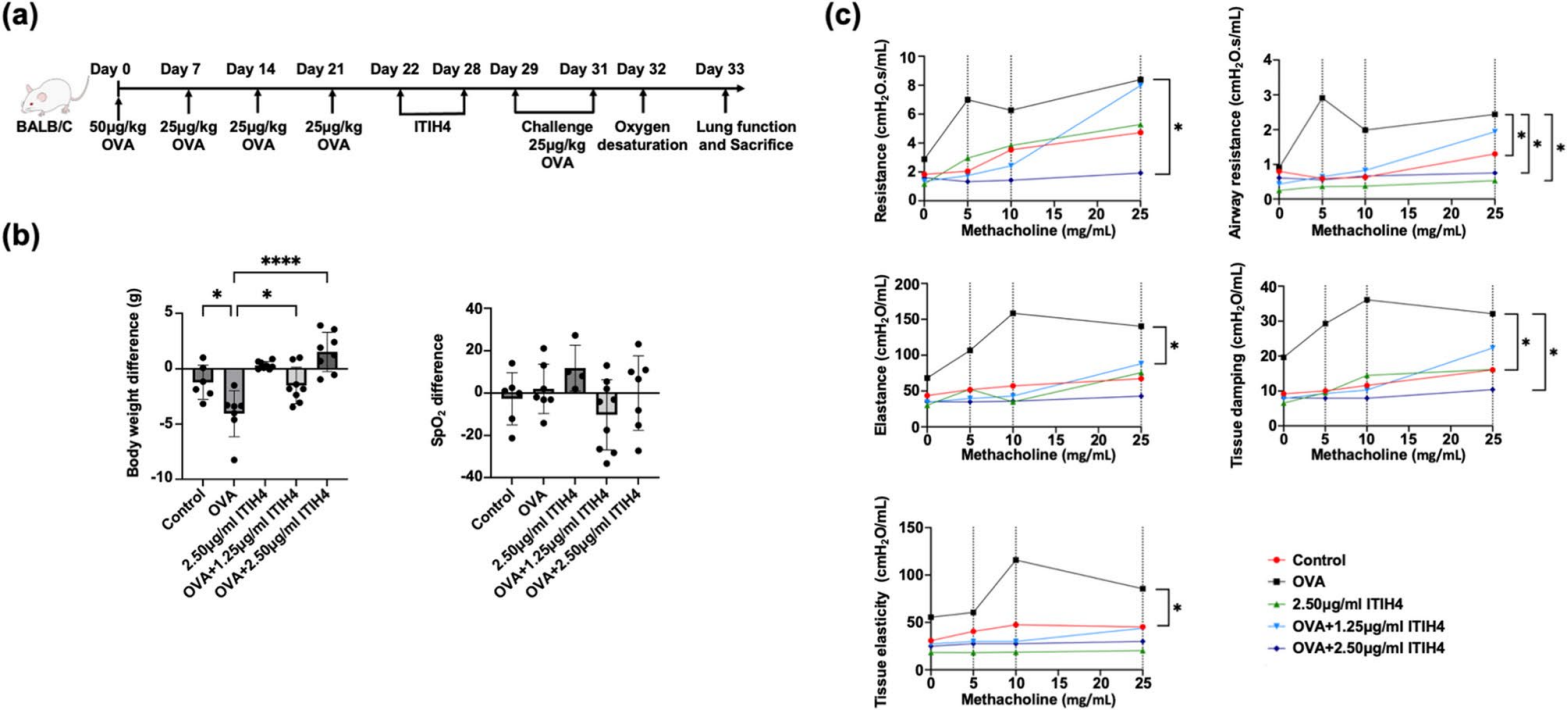


Correct Fig. 1.